# Screening, Identification, and Fermentation Optimization of the Antagonistic Actinomycete Strain TCS21-117 Against *Botrytis cinerea*

**DOI:** 10.3390/microorganisms13020379

**Published:** 2025-02-09

**Authors:** Fei Chen, Xuan Hu, Ziyang Hong, Jing Duan, Sha Zhou, Jie Chen, Dan Wang, Haiping Lin

**Affiliations:** State Key Laboratory of Subtropical Silviculture, Collaborative Innovation Center of Green Pesticide, National Joint Engineering Laboratory of Biopesticide Preparation, School of Forestry and Biotechnology, Zhejiang A & F University, Hangzhou 311300, China; 2021102031009@stu.zafu.edu.cn (F.C.);

**Keywords:** *Botrytis cinerea*, antagonistic actinomycetes, screening, identification, fermentation optimization

## Abstract

Biological control is considered one of the most important methods for preventing and controlling the worldwide fungal disease gray mold, caused by *Botrytis cinerea*. Among the various agents used in biological control, actinomycetes represent a significant group of microorganisms that offer valuable resources for biocontrol strategies. In this study, a total of 132 actinomycetes, belonging to four genera (*Streptomyces*, *Kitasatospora*, *Amycolatopsis*, and *Nocardia*), were isolated from soil. Among the five media tested, ISP-2 and GS NO.1 media were found to be highly suitable for isolating actinomycetes. It is worth mentioning that the strain TCS21-117 displayed significant inhibitory effects against *Botrytis cinerea* and nine other pathogenic fungi. The strain TCS21-117 was identified as *Streptomyces roietensis* by its morphological characteristics and phylogenetic analysis of the 16S rRNA gene. The optimum culture conditions for the strain TCS21-117 were a potato dextrose broth medium at an initial pH of 8.0, a liquid volume of 125 mL in a 250 mL flask, 180 r·min^−1^ at 28 °C, and an inoculum size of 1% for 7 days. Under these conditions, the inhibition rate against *Botrytis cinerea* was 93.31%, a significant increase (31.98%) as compared to the control. Notably, the antifungal compounds produced by the strain TCS21-117 exhibited strong stability across a range of temperatures, pH levels, and durations of storage and UV irradiation. This study showed that the *Streptomyces roietensis* strain TCS21-117 had strong inhibitory activity against *Botrytis cinerea* under optimized fermentation conditions, enriching the microbial resources for gray mold control.

## 1. Introduction

The genus *Streptomyces* was first proposed by Waksman and Henrici in 1943 [1] and is recognized to be one of the largest taxa among bacteria, serving as a rich source of secondary metabolites with diverse chemical structures and biological activities, particularly antibiotics [2]. It is estimated that over 74% of commercial antibiotics are produced from the genus *Streptomyces* [3]. As of writing, 738 species of *Streptomyces* have been validly published and accurately named (www.bacterio.net/streptomyces.html, (accessed on 2 February 2025)). Members of the genus *Streptomyces* represent a vast group of aerobic, Gram-positive bacteria characterized high DNA guanine and cytosine (G + C) content (69–78%) and a filamentous morphology akin to fungi [4,5]. A key morphological feature of this genus is the formation of long vegetative and aerial mycelia that differentiate into chains of single-celled spores [6]. Some strains are capable of producing a wide variety of pigments. They are widely distributed across various environments, including soil, animals, plants, oceans, salt lakes, and volcanoes, with a higher prevalence in soil compared to other habitats [7].

*Botrytis cinerea*, commonly known as gray mold, was ranked number 2 in a survey of the international community’s top 10 fungal plant pathogens [8]. Presently, 38 species of *Botrytis* have been formally recognized [9]. A distinguishing feature of *B. cinerea* compared to other plant pathogens is its exceptionally broad host range, capable of infecting over 1400 species of cultivated plants, particularly dicotyledonous species. Infestation can occur from the seedling stage to product ripening, presenting significant challenges to agricultural production. Annual economic losses due to *B. cinerea* are estimated to exceed USD 10 billion globally [10]. Moreover, most species and isolates of *Botrytis* exhibit a complex life cycle and flexible infection modes [11]. Infection can occur not only through the production of spores and mycelium but also via the formation of sclerotia during overwintering [12].

Chemical control is the most widely used and effective method for preventing and managing *Botrytis cinerea*, but one of its major drawbacks is the development of more aggressive resistant strains [13]. Additionally, chemical pesticides can be toxic to various organisms, including birds, fish, beneficial insects, and non-target plants, as well as impacting air, water, soil, and crops [14]. For instance, in the US, atrazine has been detected in approximately 75% of stream water samples, posing risks to aquatic ecosystems and drinking water quality [15]. Furthermore, there are approximately 385 million instances of unintentional acute pesticide poisoning each year, resulting in approximately 11,000 fatalities [16]. Therefore, the development of methods that complement chemical control, such as the use of non-pathogenic microorganisms as biological control agents (BCAs), is increasingly viewed as a promising alternative [17].

Building on the challenges posed by chemical control methods for managing *Botrytis cinerea*, this study evaluated 132 strains of actinomycetes for their antifungal activities against the pathogen. Among them, the strain TCS21-117 demonstrated notable antagonistic activity against *B*. *cinerea*. For further study, a series of assays, including broad-spectrum antifungal activity, polyphasic taxonomy, and fermentation optimization assays, were performed, which can enrich the beneficial microbial resource bank for biocontrol, and are expected to present an effective biological agent to control plant diseases.

## 2. Materials and Methods

### 2.1. Sample Collection and Pretreatment

In this study, 60 soil samples were collected from three provinces in southern China, namely Jiangsu, Zhejiang, and Shanghai (Table S1). Soil samples were taken exclusively from areas dominated by perennial herbs with a neutral-to-acidic pH at a sampling depth of 0 to 5 cm. Then, the collected soil samples were air-dried at room temperature for two weeks [18] to reduce the moisture content, inhibit the growth of non-actinomycete microorganisms, and simultaneously promote the release of spores. Once completely dried, the samples were then ground into a powder using a mortar and pestle and thoroughly mixed and passed through a 2 mm sieve to remove visible stones, roots, and other plant debris [19]. For each collected sample, a 10 g soil sample was heated at 100 °C for 1 h to facilitate the germination of actinomycete spores [20].

### 2.2. Isolation and Preservation of Actinomycetes

Actinomycetes were isolated using the serial dilution and spread plate technique [21]. Briefly, 1 g of each heated soil sample was added to 9 mL of distilled water and incubated in an orbital shaker incubator at 50 °C with shaking for 30 min at 150 r·min^−1^. The resulting solution was serially diluted up to 10^−5^ with sterile water, and 100 µL of each dilution was spread on five different media, PDA, GS NO.1, HVA, ISP-2, and GYM supplemented with nalidixic acid (25 mg·L^−1^), nystatin (50 mg·L^−1^), and K_2_Cr_2_O_7_ (50 mg·L^−1^) to inhibit the growth of Gram-negative bacteria and fungi [22]. The compositions of these media are listed in Table S2. The plates were inverted and incubated in a constant-temperature incubator at 28 °C for 7 to 10 days, with periodic monitoring for the growth of actinomycetes. Repeated streaking on fresh media plates and storage under the same conditions allowed for the purification of colonies exhibiting distinct morphological features of actinomycetes (size, aerial and substrate mycelium, powdery or leathery appearance, color, pigment production) [23]. After purification, all isolates were numbered and preserved as spore suspensions in glycerol (20%, *v*/*v*) at −80 °C for further use.

### 2.3. DNA Extraction and 16S rRNA Gene Amplification for Identification of Isolates

Molecular identification of isolates was performed through 16S rRNA gene sequencing. Biomass for molecular biological studies was prepared by growing each strain in liquid ISP-2 medium at 28 °C for a minimum of 3 days with constant agitation at 180 r·min^−1^ [24]. Total genomic DNA was extracted using a Rapid Bacterial Genomic DNA Isolation Kit (B518225, Sangon Biotech, Shanghai, China) following the manufacturer’s protocol. PCR amplification of the 16S rRNA gene was conducted using the universal primers (27F: 5′-AGTTTGATCCTGGCTCAG-3′; 1492R: 5′-ACGGCTACCTTGTTACGACTT-3′) [25]. The 25 µL PCR mixture consisted of 10.5 µL ddH_2_O, 12.5 µL Premix Taq, 1 µL DNA template, 0.5 µL 27F, and 0.5 µL 1492R. The PCR was carried out under the following conditions: initial denaturation at 95 °C for 5 min followed by 33 denaturation cycles at 95 °C for 3 min, alignment at 58 °C for 30 s, extension at 72 °C for 90 s, and a final extension at 72 °C for 5 min. The quality of the extracted DNA and PCR amplicons was evaluated by running the template on 1% agarose gel for 45 min. The purified products were sent to Qingke Biotechnology Co., Ltd. (Hangzhou, China) for sequencing, and the resulting 16S rRNA gene sequences were analyzed using BLAST with the EzBioCloud database to determine their similarity with type strains.

### 2.4. Screening of Actinomycetes for Antifungal Activity

To test the antifungal activity of actinomycetes against *Botrytis cinerea*, the plate confrontation technique was performed in a Petri plate containing PDA medium in vitro following the methodology described by Zhang et al. [26] with a few adjustments. The isolated actinomycetes were inoculated on ISP-2 medium at 28 °C for 7 days, while *B. cinerea* was cultured on PDA medium at 22 °C for 5 days. For each repetition, the target fungus cake (7 mm) was punched out and placed in the center of the PDA plate. Similar sized cakes of four different actinomycete strains were placed around the pathogen at a distance of 2 cm. A plate only inoculated with the pathogen cake served as the control. The plates were then incubated at 25 °C in the dark until the pathogenic fungus fully covered the control plate. All isolates were tested in three independent replicates, and the antagonistic strain was selected for further studies.

### 2.5. Fermentation Process

Fermentation of the antagonistic strain was performed in two stages: seed growth and production of active antifungal substances [27]. Firstly, the selected strain was cultured on plates of ISP-2 medium at 28 °C for 7 days. Then two spore disks (7 mm) were used to inoculate 75 mL GS.1 liquid medium (pH 7.2~7.4) using a 250 mL flask with shaking (180 r·min^−1^) at 28 °C for 2 days. In the second stage, 1% (*v*/*v*) of the seed culture was transferred into 250 mL flask containing 75 mL of SLM medium (pH 7.2~7.4) and incubated at 28 °C in a rotatory shaker at a speed of 180 r·min^−1^ for 7 days. After fermentation, the broth was centrifuged at 12,000 r·min^−1^, 4 °C for 10 min, and the supernatant was collected and filtered through a 0.22 μm membrane filter (Nylon 66) to obtain cell-free broth [28], which was then stored at 4 °C for further experiments.

### 2.6. Assay of a Broad-Spectrum Antifungal Activity of Strain TCS21-117 and Its Supernatant

To test the broad-spectrum antifungal activity of stain TCS21-117 and its fermentation supernatant, 10 different pathogenic fungi were selected: *Botrytis cinerea*, *Botryosphaeria dothidea*, *Corynespora cassiicola*, *Colletotrichum gloeosporioides*, *Fusarium graminearum*, *Fusarium oxysporum*, *Fusarium solani*, *Phytophthora capsica*, *Rhizoctonia solani*, and *Sclerotinia sclerotiorum*. These fungi were obtained from the Collaborative Innovation Center of Green Pesticide, National Joint Engineering Laboratory of Biopesticide Preparation, College of Forestry and Biotechnology, Zhejiang A & F University, Hangzhou, China, and were cultured on PDA medium for 5~7 days before use.

The antagonistic effects of strain TCS21-117 were determined using the dual culture assay [29]. A 7-mm-diameter disk of strain TCS21-117 was inoculated onto one side of the PDA plate, while a pathogenic fungi cake of the same diameter was placed on the opposite side, maintaining a distance of 4 cm. A plate containing only the pathogenic fungus served as the control. The antagonistic belt (inhibition zone) was measured by determining the distance between the edge of the fungal mycelium and the actinomycete cake.

The inhibitory effects of active antifungal substances on the mycelial growth of pathogenic fungi were measured using the mycelial growth rate method [30]. A mycelial disk (7 mm) of pathogenic fungi was inoculated at the center of autoclaved PDA medium containing a final supernatant concentration of 1:10 (*v*/*v*), with an equivalent amount of uninoculated medium added as the control. The cross-over method [31] was used to measure colony diameter in two perpendicular directions to obtain an average value. The inhibition rate of mycelium growth was calculated according to the description of Li et al. (2021) [32] using the following formula:

$$\mathrm{Inhibition}\;\mathrm{rate}\;(\%)\;=\frac{D_{c}-D_{t}}{D_{c}-0.7}\times 100$$

where D_c_ is the mycelial growth diameter of the blank control, and D_t_ is the mycelial growth diameter of the treatment. The diameter of the mycelial cake is 0.7 cm. All experiments were conducted in triplicates.

### 2.7. Polyphasic Taxonomic Study of Strain TCS21-117

The cultural attributes of strain TCS21-117 were determined on 10 different media plates: ISP-1~6, GS NO.1, CDA, NA, and PDA, all adjusted to pH 7.2~7.4. The color of aerial mycelia, substrate mycelia, and soluble pigments was determined visually by comparing them with chips from the ISCC-NBS centroid color charts [33].

The morphological characteristics of strain TCS21-117 were observed using the plate insert method (Figure S1) [34]. Briefly, strain TCS21-117 was streaked on a PDA plate, and a sterilized coverslip was placed at an angle of 45° using tweezers, followed by incubation at 28 °C for 7 days. After incubation, the coverslip was carefully removed from the medium, and the morphological features of aerial mycelium, substrate mycelium, and spores were examined with LM (Leica DM4 B, Wetzlar, Germany) and SEM (PW-100-011). Samples for SEM were prepared as described by Fatima et al. [35].

The temperature, pH, and NaCl tolerance for growth were evaluated using the methods described by Zhang et al. [36]. Utilization of sole carbon and nitrogen sources was determined following the methods of [37]. The catalase test, starch hydrolysis, coagulation and peptonization of milk, gelatin liquefaction, decomposition of cellulose, the degradation of Tween 40 (all at 1% *v*/*v*), hydrogen sulfide production, and melanin production were examined as described previously [38].

Genomic DNA extraction, amplification, and 16S rRNA gene sequencing were performed as described above. Alignments of multiple 16S rRNA sequences of closely related members of the genus *Streptomyces* with validly published names and sequence similarity calculations were carried out using the EzTaxon-e server [39]. Phylogenetic trees were constructed using the NJ, ML, and MP methods using the MEGA V.11.0 program [40]. The robustness of the topology of the phylogenetic trees was evaluated through bootstrap analysis with 1000 re-samplings [41], using the 16S rRNA gene of *Nocardia aobensis* strain IFM 0372T as an outgroup to root the tree [42].

### 2.8. Fermentation Optimization of Strain TCS21-117

Strain TCS21-117 was incubated in five different liquid media (GS NO.1, PDB, GYM, SLM, and MDP), following the fermentation process described previously. The compositions of these media are listed in Table S3, and the pH values were adjusted to 7.2 to 7.4. The antifungal activity of the various fermentation broths was measured according to the method described above. The medium showing the highest antifungal activity was selected as the base medium for the study of fermentation conditions.

The fermentation conditions of strain TCS21-117 were optimized to enhance its antifungal activity against *Botrytis cinerea* [43,44]. The optimization process began with varying the inoculum size, where the seed culture was introduced at concentrations of 1%, 2%, 3%, 4%, and 5% (*v*/*v*) into the optimal medium and incubated at 28 °C with shaking at 180 rpm for 7 days. Subsequently, the working volume was assessed by filling 250 mL Erlenmeyer flasks with 50, 75, 100, 125, and 150 mL of the medium, incubating under the same conditions. The fermentation time was explored by incubating cultures for 1, 3, 5, 7, and 9 days, while varying the fermentation temperature from 22 to 34 °C (at intervals of 3 °C) at a constant agitation of 180 r·min^−1^. Finally, the initial pH of culture medium was optimized by incubating the cultures at pH values ranging from 4.0 to 12.0 in 2.0 increments at 28 °C. Each treatment was performed in triplicate. The antifungal activity was evaluated using the mycelial growth rate assay to systematically optimize the fermentation conditions for strain TCS21-117. A control group was maintained for each factor tested, utilizing the standard conditions established in our laboratory (28 °C, 180 r·min^−1^, pH 7.2–7.4, 1% inoculum size, and 7 days).

Based on the results of the single-factor experiments described above, an orthogonal experimental design was employed to further optimize the fermentation conditions, using the antifungal activity of the fermentation broth as the evaluation index. A four-factor, three-level orthogonal experiment was conducted according to the experimental design shown in Table 1.

### 2.9. Evaluation of the Stability of TCS21-117 Fermentation Broth

The stability of the fermentation broth produced by strain TCS21-117 was evaluated by assessing its antifungal activity under various treatment conditions, including temperature, pH, storage, and UV irradiation [45]. For thermal stability, fermentation broth was heated in sterilized Erlenmeyer flasks for 20 min at temperatures of 50 °C, 60 °C, 70 °C, 80 °C, 90 °C, and 100 °C and autoclaved at 121 °C. Untreated fermentation broth was used as a control, and each treatment repeated in triplicate. For pH stability, the pH of the fermentation broth was adjusted to 4.0, 6.0, 8.0, 10.0, and 12.0. After allowing the samples to stand for 24 h, the pH was readjusted to its original value, with untreated broth at its original pH value used as a control. Each treatment was also repeated in triplicate. Regarding storage stability, 50 mL aliquots of the fermentation broth were placed in sterile sealed bottles and stored at 4 °C in a refrigerator or at room temperature. The antifungal activity of the aliquots was determined at 30, 60, and 90 days, with each treatment repeated in triplicate. For UV stability, the fermentation broth was placed in sterile culture dishes and irradiated for 1, 3, 5, 7, 9, 11, and 13 h using a UV lamp positioned 10 cm away. Non-irradiated fermentation broth was used as a control, and each treatment repeated in triplicate.

### 2.10. Statistical Analysis

All experimental data were obtained from at least three independent experiments and are expressed as the mean ± standard deviation (SD). The data were analyzed using IBM SPSS^®^ Statistics (Version 25.0, Armonk, NY, USA). Statistically significant differences between means were evaluated using one-way analysis of variance followed by Dunnett’s post hoc comparisons test with a significance level set at *p* < 0.05.

## 3. Results

### 3.1. Isolation and Identification of Actinomycetes

**Figure 1:**
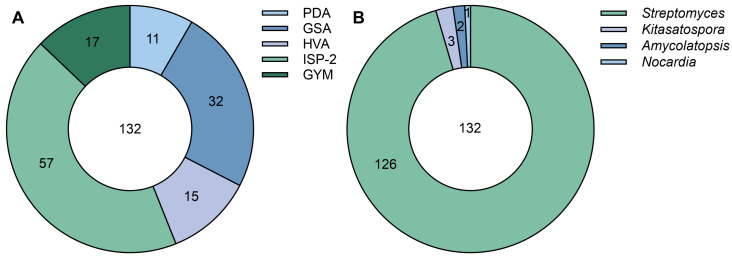
Species and distribution of the isolated actinomycetes. (**A**) The number of isolates on different isolation media; (**B**) The number of isolates per genus obtained.

A total of 132 actinomycetes with different morphological characteristics were successfully isolated from 60 soil samples using the gradient dilution separation method. Among these, 11, 32, 15, 57, and 17 strains were isolated from PDA, GS NO.1, HVA, ISP-2, and GYM media plates, respectively (Figure 1A). A variety of colors of aerial mycelia were observed across different strains, including white, yellow, green, black, etc. Notably, special pigments were observed in four strains (Figure S2): TCS-007 (light pink), TCS21-026 (dark red), TCS21-057 (dark purple), and in TCS21-079 (light green). Preliminary identification of these 132 strains was conducted based on 16S rRNA gene sequencing, which revealed that all strains were classified into four genera of actinomycetes: *Streptomyces*, *Kitasatospora*, *Amycolatopsis*, and *Nocardia*. Remarkably, *Streptomyces* was the most frequently isolated genus, comprising 126 isolates (95%) belonging to 47 species, followed by *Kitasatospora* (three isolates), *Amycolatopsis* (two isolates), and *Nocardia* (one isolate) (Figure 1B). On the other hand, the 16S rRNA gene sequences of strains TCS21-010, TCS21-030, TCS21-031, TCS21-054, and TCS21-117 showed 98.48%, 98.79%, 98.69%, 98.92%, and 98.58% identity to *S. gossypiisoli* TRM 44567^T^ (MN548415), *S. pseudovenezuelae* DSM 40212^T^ (KQ948163), *S. pratensis* ch24^T^ (JQ806215), *S. cyaneochromogenes* MK-45^T^ (MG324360), and *S. roietensis* WES2^T^ (KX394336), respectively (Table 2). These strains are likely to represent new species, and taxonomic studies are underway.

### 3.2. Antifungal Activity of Isolates Against B. cinerea

**Figure 2:**
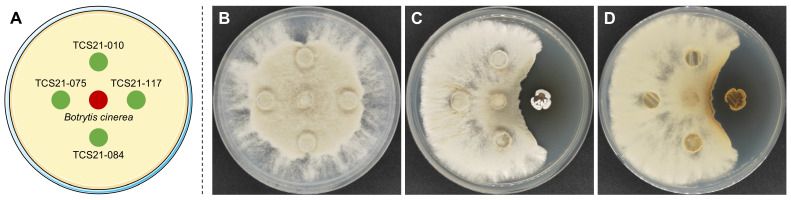
Antagonistic activity of strain TCS21-117 against *B. cinerea*. (**A**) Schematic diagram of the experiment; (**B**) Control group; (**C**) Front side of the experimental group; (**D**) Reverse side.

The actinomycetes with antifungal activity against *B. cinerea* were screened using the plate confrontation method. Out of a total of 132 actinomycete isolates, only 6 strains of *Streptomyces* showed antagonistic activity against *B. cinerea*. Notably, the *Streptomyces* sp. TCS21-117 strain showed high activity against *B. cinerea* (Figure 2C). The distinctly larger inhibition zone seen in this study strongly suggests the release of a greater amount of antibiotic, which contributed to the observed inhibition.

### 3.3. Antifungal Spectrum of Strain TCS21-117

**Figure 3:**
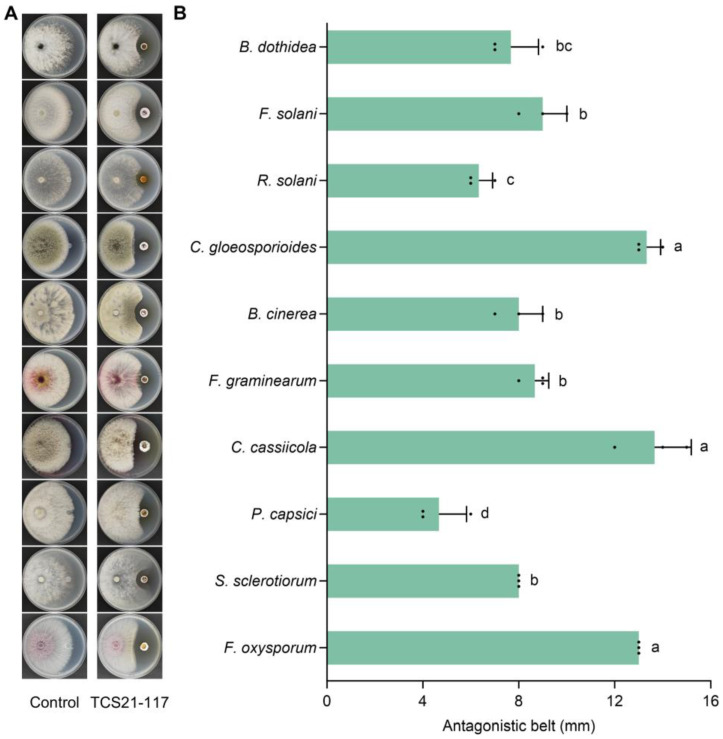
Antifungal activity of strain TCS21-117 against the tested fungi. (**A**) The phenotype of colony growth; (**B**) Antagonistic belt. Data are the mean value ± standard error of three replicates, and bars with different letters (a–d) are significantly different in the LSD test.

The antifungal ability of strain TCS21-117 was determined using the plate confrontation method. As shown in Figure 3A, strain TCS21-117 significantly inhibited the growth of all 10 tested pathogens, and the inhibition zone (antagonistic belt) ranged from 4.67 ± 1.15 mm (*P. capsici*) to 13.67 ± 1.53 mm (*C. cassiicola*) (Figure 3B), indicating a broad antifungal spectrum for strain TCS21-117.

**Figure 4:**
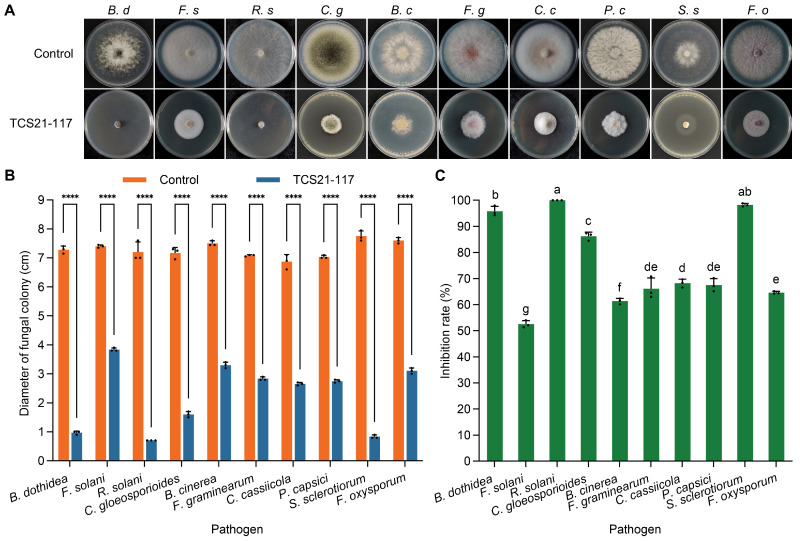
The broad-spectrum antifungal activity of TCS21-117 fermentation broth against different pathogenic fungi. (**A**) The phenotype of colony growth; (**B**) Colony diameter; (**C**) Inhibition rate. **** represents the significant differences between the control and treatment groups at *p* < 0.0001 level (*t*-test). Bars with different letters mean significant differences at *p* < 0.05 level.

To further elucidate the antifungal activity of TCS21-117, its fermentation supernatant was collected to detect the inhibitory activity of pathogenic fungi using the mycelial growth rate method. The inhibition rates for each indicator pathogen demonstrated the broad-spectrum antifungal activity of TCS21-117 fermentation broth (Figure 4A−C). *R. solani* was the most sensitive to the TCS21-117 fermentation broth, exhibiting an inhibition percentage of 100.00 ± 0.00%. Other pathogens, including *B. dothidea*, *F. solani*, *C. gloeosporioides*, *B. cinerea*, *F. graminearum*, *C. cassiicola*, *P. capsica*, *S. sclerotiorum*, and *F. oxysporum*, also showed sensitivity to the TCS21-117 fermentation broth, with inhibition rates of 95.82 ± 1.91%, 52.52 ± 1.38%, 86.24 ± 1.51%, 61.33 ± 1.12%, 66.11 ± 4.13%, 68.21 ± 1.57%, 67.48 ± 2.51%, 98.21 ± 0.61%, and 64.61 ± 0.51%, respectively. The inhibition function of secondary metabolites produced by TCS21-117 significantly inhibited filamentous pathogenic fungi, especially *R. solani*, *S. sclerotiorum*, and *B. dothidea*. Due to the strong antagonistic activity against the tested pathogenic fungi, strain TCS21-117 was selected for further studies on its taxonomic position through various methods.

### 3.4. Characterization of Strain TCS21-117

The cultural characteristics of TCS21-117 on various media plates are presented in Table 3 and Figure S3. Strain TCS21-117 exhibited an excellent growth phenotype on ISP-2 and PDA media plates, good growth on ISP-1, ISP-3, ISP-6, GS NO.1, CDA, and NA media plates, and average growth on ISP-4 and ISP-5 media plates. The colony colors varied from white to yellowish white, while substrate mycelium varied from light yellow to yellowish brown. The light-yellow soluble pigment was produced on ISP-1-4, GS NO.1, NA, and PDA plates.

**Figure 5:**
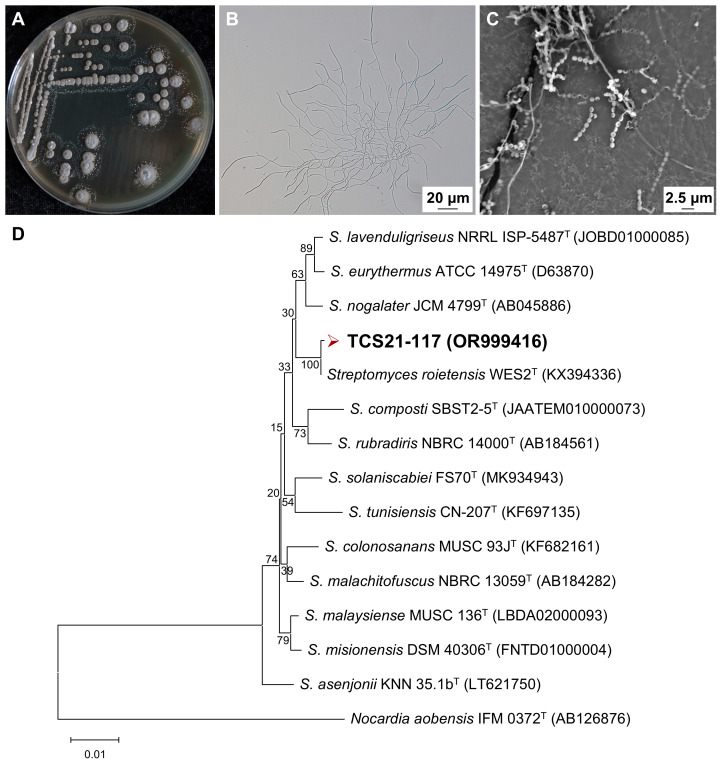
Taxonomy of *Streptomyces* sp. TCS21-117 based on morphological characteristics and phylogenetic analyses. (**A**) Colony morphology (PDA, 28 °C, 7 days); (**B**) Mycelium morphology (PDA, 28 °C, 2 days, OM, 400×, Bar, 20 μm); (**C**) Spore chain morphology (PDA, 28 °C, 7 days, SEM, 5500×, Bar, 2.5 μm); (**D**) Phylogenetic tree based on 16S rRNA sequences (NJ, Bar, 0.01 nucleotide substitutions per site).

Morphological observation of strain TCS21-117 grown on PDA medium revealed typical characteristics of the genus *Streptomyces*. The strain formed leathery colonies with abundant spores, showing light-gray aerial mycelia and light-yellow substrate mycelia with yellow diffusible pigments (Figure 5A). The aerial mycelia were difficult to pick up, elongated, and multi-branched mycelia were visible under an optical microscope (Figure 5B). The aerial hyphae differentiate into long chains of prespore compartments, which then develop thick spore walls. Under the scanning electron microscope, the mature spores were found to be oval to round in shape, with a smooth surface and with dimensions of approximately (0.7~1.0) × (1.3~1.5) μm (Figure 5C).

For the physiological and biochemical characterizations of TCS21-117, as shown in Table 4, the growth of TCS21-117 was observed at the temperature range of 19~50 °C (optimum at 28 °C), the pH range of 4.0~12.0 (optimum at 7.0), and the NaCl tolerance range of 0~7% (optimum 2%). Strain TCS21-117 could utilize soluble starch, D-lactose, D-maltose, D-fructose, D-glucose, L-rhamnose, and L-arabinose as sole carbon sources but not D-sorbitol, D-mannitol, or sucrose. Strain TCS21-117 could utilize NaNO_3_, KNO_3_, peptone, yeast powder, and alanine as sole nitrogen sources but not (NH_4_)_2_SO_4_, arginine, or urea. The above results showed that strain TCS21-117 broadly utilized carbon and nitrogen sources. Biochemical characterizations revealed that strain TCS21-117 could produce catalase but was negative for H_2_S production. Melanin pigment was detected on the ISP-6 medium. It was positive in tests for Tween 40, gelatin liquefaction, coagulation, peptonization of milk, starch hydrolysis, and cellulose hydrolysis.

To further classify the strain TCS21-117, its 16S rRNA gene was amplified, and the 1417 bp product fragment was sequenced and deposited with the accession number OR999416 in the GenBank database. Phylogenetic analysis based on 16S rRNA gene sequences revealed that strain TCS21-117 belongs to the genus *Streptomyces,* displaying the highest 16S rRNA gene sequence similarity value to *S. roietensis* WES2^T^ (GenBank accession number: KX394336; 98.58%), followed by *S. colonosanans* MUSC 93J^T^ (GenBank accession number: KF682161; 98.07%) (Table 5). The result of phylogenetic analysis using the neighbor-joining method (Figure 5D), based on 16S rRNA gene sequences, indicated that strain TCS21-117 clustered with the type strain *S. roietensis* WES2^T^ at a bootstrap value of 100%. This finding was further supported by the results obtained with the maximum-likelihood and maximum-parsimony tree-making algorithms (Figure S4).

Combining the cultural phenotypes, morphological observations, physiological and biochemical characteristics, and phylogenetic analysis of 16S rRNA gene sequences, strain TCS21-117 belongs to the genus *Streptomyces* and it has the highest similarity to *S. roietensis*.

### 3.5. Optimization of Fermentation Conditions

**Figure 6:**
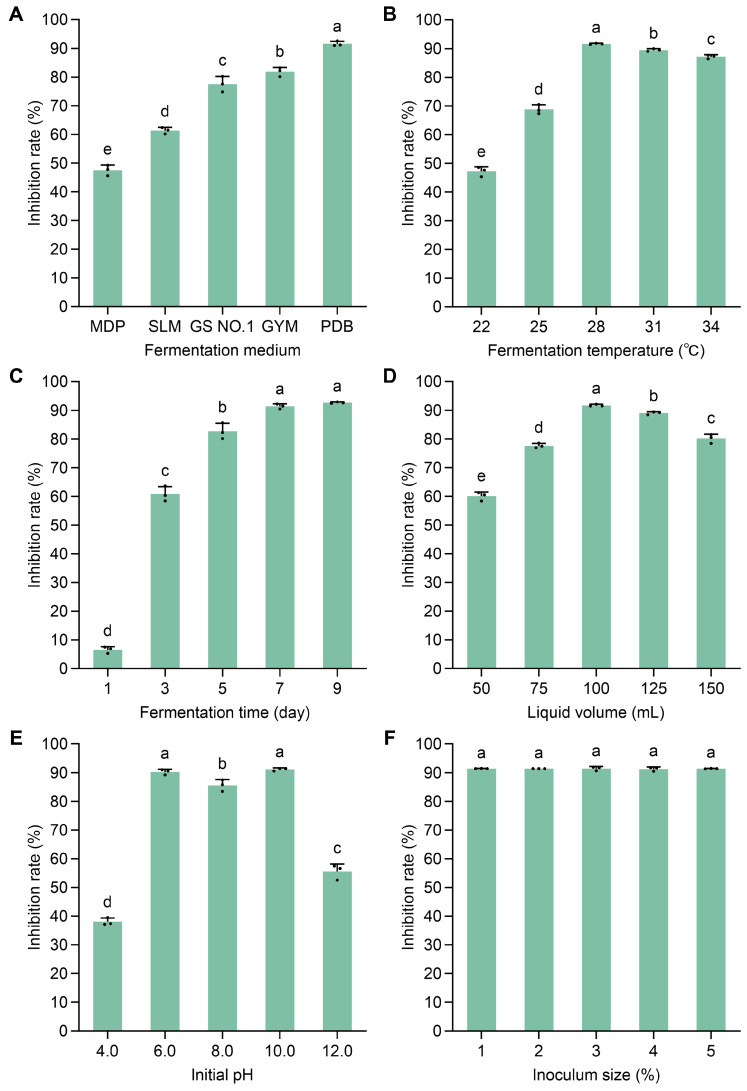
Optimization of culture conditions for antifungal activity of *Streptomyces* sp. TCS21-117 against *B. cinerea*. (**A**) Fermentation media; (**B**) Temperature (°C); (**C**) Time (day); (**D**) Liquid volume (mL); (**E**) Initial pH; (**F**) Inoculum size (%). Bars represent mean inhibition rates ± standard deviation. Different letters above bars indicate statistically significant differences (*p* < 0.05) as determined by Duncan’s multiple range test.

Five fermentation media with diverse nutrient compositions were evaluated to enhance the antifungal activity of the antagonistic strain TCS21-117. As shown in Figure 6A, strain TCS21-117 cultured in PDB medium exhibited the highest inhibitory activity (91.56 ± 0.87%) against *B. cinerea*, significantly surpassing the results obtained in the other four media. Consequently, PDB medium was selected as the basal fermentation medium for further optimization of fermentation conditions.

Strain TCS21-117 was cultured under various fermentation conditions, and the results indicated that fermentation temperature, time, liquid volume, and initial pH all affected the inhibition rate of the fermentation broth. As shown in Figure 6B, it can be seen that the inhibition rate of the fermentation broth increased at first and then decreased. The highest inhibition rate was observed at 28 °C, reaching 91.57%, which was significantly higher than other temperatures. As shown in Figure 6C, the inhibition rate increased from 1 day, with the greatest inhibition of 91.48% seen at 7 days; this rate was not significantly different from that at 9 days but was significantly higher than at 1, 3, and 5 days. As shown in Figure 6D, the highest inhibition rate of 91.40% was achieved with a medium volume of 100 mL in a 250 mL flask, significantly different from other liquid volumes. As shown in Figure 6E, the highest inhibition rates were observed at pH 6.0 and 10.0. As shown in Figure 6F, there was no significant effect on the inhibition rate caused by the treatments of different inoculum sizes.

Based on the result of single-factor experiments, four main influential factors were identified: temperature, time, initial pH, and liquid volume. To optimize the fermentation system, an L_9_(3^4^) orthogonal design was employed. From the range analysis in Table 6, it can be seen that the effect of the various factors on antifungal activity was ranked as B > C > A > D (time > initial pH > temperature > liquid volume). The optimal combinations were identified as B_2_C_2_A_2_D_3_ and B_2_C_1_A_3_D_3_ according to the K value and the orthogonal results, respectively. Verification tests showed that the inhibition rate of fermentation broth under optimal conditions reached up to 93.31%, which was 31.98% higher than before optimization (SLM medium, 61.33%).

### 3.6. Stability of Fermentation Broth

**Figure 7:**
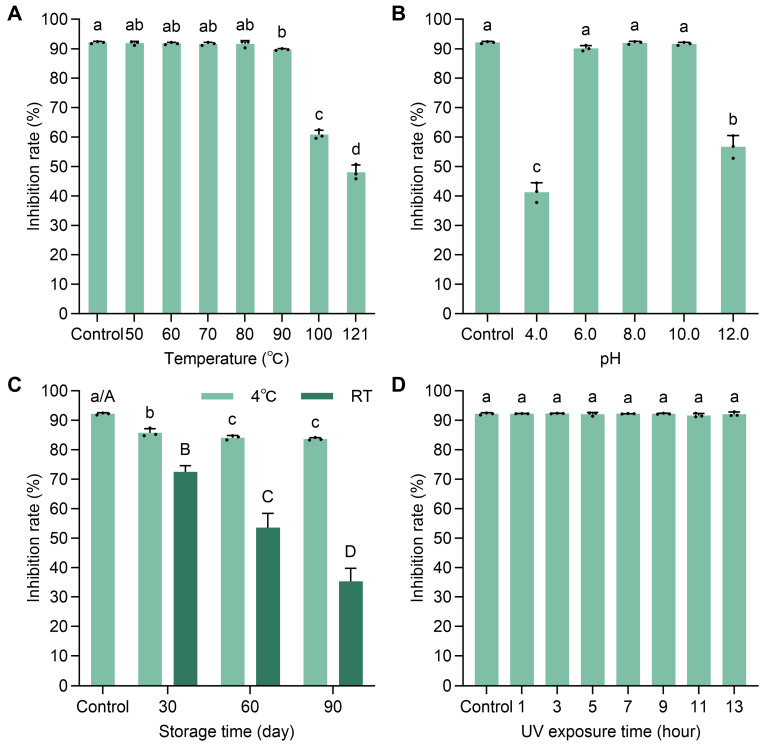
Stability of antifungal compounds produced by *Streptomyces* sp. TCS21-117 under various environmental conditions. (**A**) Temperature; (**B**) pH level; (**C**) Storage time; (**D**) UV exposure time. Data are a mean value ± standard error of three replicates, and bars with different letters ((a–d) and (A–D)) are significantly different in the LSD test.

The stability of the antifungal compounds in strain TCS21-117’s fermentation broth was evaluated under varying temperatures, pH levels, storage conditions and UV exposure times. The fermentation broth maintained its antifungal activity at 50 °C, 60 °C, 70 °C, and 80 °C, with minimal reduction at 90 °C for 20 min. However, excessive temperature or prolonged heat treatment significantly reduces its antifungal activity (Figure 7A). Antifungal activity remained stable between pH 6.0 and 10.0, and acid treatment was more likely to cause the inactivation of the fermentation broth (Figure 7B). The fermentation broth demonstrated greater stability when stored at 4 °C compared to room temperature (Figure 7C). Additionally, antifungal activity showed resilience to UV exposure, with no significant changes observed (Figure 7D).

## 4. Discussion

The discovery of novel bioactive compounds is an ongoing process [46]. One of the more effective methods for discovering novel metabolites from microorganisms is through the isolation of new microbial species [47]. Soil, as the natural culture medium, contains a diverse array of microorganisms that produce biologically active compounds. However, less than 1% of the microbial community can be cultivated by using conventional culturing techniques, leaving the remaining 99% of microorganisms unexplored [48]. New species of *Streptomyces* may possess unique characteristics and produce specific secondary metabolites.

In this study, a total of 132 strains of actinomycetes were isolated from 60 soil samples using five kinds of media. However, the 16S rRNA gene sequencing showed that these isolates were mostly *Streptomyces*. This indicates that the isolation method has some limitations and requires further improvement. The literature showed that the main factors affecting the separation of actinomycetes include the different pretreatment methods and media combinations [49]. Kang et al. [50] reported that the separation effect of actinomycetes was effective when the soil samples were treated for 7 to 10 days by air drying, resulting in a greater number and more types of actinomycetes, while it significantly reduced the presence of miscellaneous bacteria. According to the study by Si et al. [51], pretreatment of air-dried soil samples with dry heat at 100 °C for 60 min facilitated the germination of actinomycete spores and increased both the number and species of actinomycetes. However, the effectiveness of heat treatment on reducing bacterial growth was not optimal. Yang et al. [52] examined the effects of different actinomycetes on inhibitors of potassium bichromate and found that potassium bichromate was a highly selective, effective, and inexpensive inhibitor that could suppress most soil fungi and bacteria but not actinomycetes. In addition, many studies have shown that ISP series media (ISP-2, ISP-4, ISP-5) exhibit high selectivity for the actinomycete group.

The plate confrontation method and the mycelial growth rate method performed in this study have been widely applied in the screening of beneficial microorganisms with biocontrol potential. In this study, strain TCS21-117 showed moderate antagonistic activity to *R. solani* on the PDA plate; however, the fermentation broth of strain TCS21-117 showed almost complete inhibition against *R. solani*. This phenomenon can be attributed to at least two factors. One reason is that the change in the culture method (solid-state and liquid) has altered the expression patterns of the secondary metabolic pathways within strain TCS21-117 [53]. Another is the difference in growth rates between fungi and actinomycetes; the growth and reproduction of actinomycetes are slower than those of fungi [21]. Consequently, it takes longer for actinomycetes to colonize and produce active substances on the plate, and the diffusion of these active substances also requires time, which may not be sufficient to inhibit pathogenic fungal growth.

Strain TCS21-117 was identified as *Streptomyces* using traditional classification methods and 16S rRNA gene sequencing technology. It was found that strain TCS21-117 was most closely related to *Streptomyces roietensis* WES2^T^ (98.58% 16S rRNA gene sequence similarity) and *Streptomyces colonosanans* MUSC 93J^T^ (98.07%) (Table 5). These closely related *Streptomyces* strains are known to produce a range of useful antibiotics. However, there are notable differences between strain TCS21-117 and its closely related strains regarding phenotypic, physiological, and biochemical properties. Strain TCS21-117 can be easily distinguished from its most closely related species by cultural characteristics, such as colony colors and diffusible pigment production. Morphological characteristics, including spore chain formation and surface ornamentation, also distinguish the isolate from its closely related strains. In addition, strain TCS21-117 can grow moderately in the presence of 7% NaCl, pH 4.0 or 12.0, and 50 °C, in contrast to its closely related strains, which cannot grow under these conditions. While strain TCS-21-117 cannot utilize D-mannitol and sucrose, *S. roietensis* WES2^T^ can [54], allowing for differentiation between the two. Furthermore, strain TCS21-117 can utilize L-rhamnose, D-lactose, D-maltose, and D-fructose, whereas *S. colonosanans* MUSC 93J^T^ cannot [55], providing another distinguishing factor. Strain TCS21-117 can hydrolyze cellulose, while *S. colonosanans* MUSC 93J^T^ cannot. Therefore, further identification is needed, including chemotaxonomic analyses and genomic studies.

Actinomycete fermentation is a complex and dynamic biological process [56]. Studies have shown that the types and yield of fermentation products are influenced not only by the performance of the strain but also by nutrients and environmental factors, such as inoculation volume, medium capacity, fermentation time, temperature, agitation rate, and initial pH [57]. In this study, the effects of different types of culture media and fermentation conditions on the antifungal activity of fermentation broth of strain TCS21-117 were analyzed using single-factor experiments and orthogonal tests. The results showed that PDB broth medium is highly suitable for cultivating strain TCS21-117, as it is simple, low-cost, and easy to promote. The ideal fermentation conditions were determined to be fermentation temperature of 28 °C, a duration of 7 days, an inoculation volume of 1%, a medium capacity of 125 mL, an agitation rate of 180 r·min^−1^, and an initial pH of 8.0. Fermentation temperature and time significantly impact the antifungal activity of strain TCS21-117. Compared with other antagonistic *Streptomyces*, other fermentation conditions such as inoculation volume had no effect on the antifungal activity of fermentation broth. This suggests that there are certain differences in the utilization of carbon and nitrogen sources and fermentation conditions among different strains belonging to the same genus. These differences may be related to the genetic, physiological characteristics, isolation sources, and the evaluation of the antifungal activity of pathogenic targets of the strains themselves [58]. Additionally, the current study found that the active compounds in the fermentation broth of actinomycete strain TCS21-117 demonstrated remarkable stability against various environment conditions, including thermal, pH level, storage, and UV light exposure time. This stability is a significant advantage for the development and application of biocontrol agents based on this strain.

The use of *Streptomyces* strain TCS21-117 as a biocontrol agent offers significant environmental advantages over chemical fungicides, which are associated with soil and water pollution, toxicity to non-target organisms, and risks to human health. In contrast, *Streptomyces*-based agents are environmentally sustainable and pose minimal ecological hazards. Studies have demonstrated that *Streptomyces* strains can reduce chemical fungicide while maintaining crop yields [59]. The fermentation broth of TCS21-117 exhibited strong antifungal activity against *B. cinerea*, underscoring its potential as an effective alternative to chemical fungicides. Furthermore, *Streptomyces* spp. are known to degrade pesticide residues, enhancing soil health and reducing environmental contamination [60]. The active compounds produced by TCS21-117 also demonstrated high stability under thermal, pH, and UV stress, supporting their suitability for field applications. Therefore, TCS21-117 represents a promising, eco-friendly alternative for sustainable agriculture. Further studies will be conducted on the isolation and characterization of bioactive compounds, and studies on antagonistic mechanisms will be carried out.

## Figures and Tables

**Table 1:** Factors and levels of orthogonal experiments.

Levels	Factors			
	A: Temperature (°C)	B: Time (Day)	C: Initial pH	D: Liquid Volume (mL)
1	26	6	6	75
2	28	7	8	100
3	30	8	10	125

**Table 2:** Taxonomic affiliation of five actinomycete strains.

Isolate No.	Length (bp)	Top-Hit Strain	Similarity (%)
TCS21-010	1468	*S. gossypiisoli* TRM 44567 ^T^	98.48
TCS21-030	1405	*S. pseudovenezuelae* DSM 40212 ^T^	98.79
TCS21-031	1439	*S. pseudovenezuelae* DSM 40212 ^T^	98.79
TCS21-054	1385	*S. cyaneochromogenes* MK-45 ^T^	98.92
TCS21-117	1417	*S. roietensis* WES2 ^T^	98.58

^T^ indicates type strain.

**Table 3:** Cultural characteristics of strain TCS21-117 on different media.

Media	Growth Situation	Color of Colony Mycelia		Diffusible Pigment
		Aerial	Substrate	
ISP-1	++	Shell white	Yellowish white	Light yellow
ISP-2	+++	White	Light yellow	Maize yellow
ISP-3	++	White	Yellowish white	Yellowish brown
ISP-4	+	Yellowish white	Creamy yellow	Light yellow
ISP-5	+	White	Grayish white	−
ISP-6	++	White	Light coffee	Dark purple
GS NO.1	++	White	Maize yellow	Light yellow
CDA	++	Milk white	Oyster white	−
NA	++	White	Maize yellow	Light yellow
PDA	+++	White	Yellowish brown	Light yellow

+++, Excellent growth; ++, Good growth; +, Average growth; −, Not observed.

**Table 4:** Physiological and biochemical characteristics of strain TCS21-117.

Characteristics	TCS21-117	Characteristics	TCS21-117
**Growth phenotypes on ISP-2**		**Nitrogen utilization**	
Aerial mycelium	White	KNO_3_	+
Substrate mycelium	Light yellow	NaNO_3_	+
**Growth conditions**		(NH_4_)_2_SO_4_	−
Growth temperature (°C)	19~50	Urea	−
pH range for growth	4.0~12.0	Peptone	+
NaCl tolerance range (*w*/*v*, %)	0~7	D-alanine	+
**Carbon utilization**		D-arginine	−
Sucrose	−	Yeast powder	+
Soluble starch	+	**Other characteristics**	
L-rhamnose	+	Catalase	+
**Carbon utilization**		**Other characteristics**	
L-arabinose	+	Tween 40	+
D-lactose	+	H_2_S production	−
D-maltose	+	Starch hydrolysis	+
D-fructose	+	Gelatin liquefaction	+
D-glucose	+	Melanin production	+
D-sorbitol	−	Cellulose hydrolysis	+
D-mannitol	−	Coagulation and peptonization of milk	+

+, positive; −, negative.

**Table 5:** Comparison between the 16S rRNA gene sequence of strain TCS21-117 (OR999416) and those of closely related type strains of *Streptomyces* species.

No.	Strain	Accession Number	Similarity (%)
1	*Streptomyces roietensis* WES2 ^T^	KX394336	98.58
2	*S. colonosanans* MUSC 93J ^T^	KF682161	98.07
3	*S. solaniscabiei* FS70 ^T^	MK934943	97.79
4	*S. composti* SBST2-5 ^T^	JAATEM010000073	97.76
5	*S. asenjonii* KNN 35.1b ^T^	LT621750	97.47
6	*S. nogalater* JCM 4799 ^T^	AB045886	97.46
7	*S. malaysiense* MUSC 136 ^T^	LBDA02000093	97.45
8	*S. lavenduligriseus* NRRL ISP-5487 ^T^	JOBD01000085	97.39
9	*S. tunisiensis* CN-207 ^T^	KF697135	97.32
10	*S. eurythermus* ATCC 14975 ^T^	D63870	97.18

^T^ indicates type strain.

**Table 6:** Results of orthogonal test of fermentation conditions.

Test Numbers	A	B	C	D	Inhibition Rate (%)
1	26	6	6	75	87.45
2	26	7	8	100	93.04
3	26	8	10	125	86.94
4	28	6	8	125	92.06
5	28	7	10	75	93.12
6	28	6	6	100	87.82
7	30	6	10	100	85.89
8	30	7	6	125	93.31
9	30	8	8	75	89.73
K1	89.14	88.47	89.53	90.10	
K2	91.00	93.16	91.61	88.92	
K3	89.64	88.16	88.65	90.77	
k1	29.71	29.49	29.84	30.03	
k2	30.33	31.05	30.53	29.64	
k3	29.88	29.38	29.55	30.26	
R	1.86	5.00	2.96	1.85	
Factors affecting	B > C > A > D				
Optimization level	B_2_C_2_A_2_D_3_				

K means the average inhibition rate of each factor at each level; k is the arithmetic mean of K; R represents the range of the average inhibition rate under each level of the same factor (range = the maximum average inhibition rate − the minimum average inhibition rate).

## Data Availability

All data generated or analyzed during this study are included in the article, its Supplementary Materials files are publicly available in repositories. The gene sequences presented in this study have been deposited in the NCBI GenBank database under accession number OR999416.

## Supplementary Materials

The following supporting information can be downloaded at: https://www.mdpi.com/article/10.3390/microorganisms13020379/s1, Figure S1: Schematic diagram of the insertion Method; Figure S2: Special pigments produced by four *Streptomyces* isolates in this study. (A) TCS21-007; (B) TCS21-026; (C) TCS21-057; (D) TCS21-079; Figure S3: The colony morphology of strain TCS21-117 on different medium plates; Figure S4: The result of phylogenetic analysis using the maximum-likelihood (A) and maximum-parsimony (B) tree-making algorithms; Table S1: Location of soil samples collection; Table S2: Media formulations for isolation of actinomycetes; Table S3: Media formulations for fermentation of strain TCS21-117.

## Abbreviations

bp	Base Pair
ISP	International *Streptomyces* Project
NBS/ISCC	National Bureau of Standards/Inter-Society Color Council

## References

[1] Waksman S.A., Henrici A.T. (1943). The nomenclature and classification of the actinomycetes. J. Bacteriol..

[2] Duangupama T., Pratuangdejkul J., Chongruchiroj S., Pittayakhajonwut P., Intaraudom C., Tadtong S., Nunthanavanit P., Samee W., He Y.W., Tanasupawat S. (2023). New insights into the neuroprotective and beta-secretase1 inhibitor profiles of tirandamycin B isolated from a newly found *Streptomyces composti* sp. nov. Sci. Rep..

[3] Bhavana M., Talluri V.P., Siva Kumar K., Rajagopal S.V. (2014). Optimization of culture conditions of *streptomyces carpaticus* (MTCC-11062) for the production of antimicrobial compound. Int. J. Pharm. Pharm. Sci..

[4] Sandoval-Powers M., Králová S., Nguyen G.S., Fawwal D.V., Degnes K., Lewin A.S., Klinkenberg G., Wentzel A., Liles M.R. (2021). *Streptomyces poriferorum* sp. nov., a novel marine sponge-derived Actinobacteria species expressing anti-MRSA activity. Syst. Appl. Microbiol..

[5] Procópio R.E., Silva I.R., Martins M.K., Azevedo J.L., Araújo J.M. (2012). Antibiotics produced by *Streptomyces*. Braz. J. Infect. Dis..

[6] Jones S.E., Elliot M.A. (2017). *Streptomyces* exploration: Competition, volatile communication and new bacterial behaviours. Trends Microbiol..

[7] Somphong A., Poengsungnoen V., Buaruang K., Suriyachadkun C., Sripreechasak P., Tanasupawat S., Phongsopitanun W. (2022). Diversity of the culturable lichen-derived actinobacteria and the taxonomy of *Streptomyces parmotrematis* sp. nov. Antonie Van Leeuwenhoek.

[8] Dean R., Van Kan J.A.L., Pretorius Z.A., Hammond-Kosack K.E., Di Pietro A., Spanu P.D., Rudd J.J., Dickman M., Kahmann R., Ellis J. (2012). The Top 10 fungal pathogens in molecular plant pathology. Mol. Plant Pathol..

[9] Ullah I., Yuan W.B., Khalil H.B., Khan H.R., Lak F., Uzair M., Abbas A., Gohari A.M., Wu H.Z. (2024). Understanding *Botrytis cinerea* infection and gray mold management: A review paper on deciphering the rose’s thorn. Phytopathol. Res..

[10] Ma J.H., Ying M.X., Lu Z.W., Guan Z.W., Zhang C.Q., Zhu X.L., Yang G.F. (2024). The resistance mechanism of B_P225F and B_H272R mutations in succinate dehydrogenase in *Botrytis cinerea*. Int. J. Biol. Macromol..

[11] Cheung N., Tian L., Liu X., Li X. (2020). The destructive fungal pathogen *Botrytis cinerea*–insights from genes studied with mutant analysis. Pathogens.

[12] Alkilayh O.A., Hamed K.E., Sayyed R.Z., Abdelaal K., Omar A.F. (2024). Characterization of *Botrytis cinerea*, the causal agent of tomato grey mould, and its biocontrol using *Bacillus subtilis*. Physiol. Mol. Plant Pathol..

[13] Shao W.Y., Zhao Y.F., Ma Z.H. (2021). Advances in understanding fungicide resistance in *Botrytis cinerea* in China. Phytopathology.

[14] Tudi M., Daniel Ruan H., Wang L., Lyu J., Sadler R., Connell D., Chu C., Phung D.T. (2021). Agriculture development, pesticide application and its impact on the environment. Int. J. Environ. Res. Public Health.

[15] Schreinemachers P., Grovermann C., Praneetvatakul S., Heng P., Loc Nguyen T.T., Buntong B., Le N.T., Pinn T. (2020). How much is too much? Quantifying pesticide overuse in vegetable production in Southeast Asia. J. Clean. Prod..

[16] Garud A., Pawar S., Patil M.S., Kale S.R., Patil S. (2024). A scientific review of pesticides: Classification, toxicity, health effects, sustainability, and environmental impact. Cureus.

[17] Bolívar-Anillo H.J., Garrido C., Collado I.G. (2020). Endophytic microorganisms for biocontrol of the phytopathogenic fungus *Botrytis cinerea*. Phytochem. Rev..

[18] Zhao S.S., Cheng M., Lin C.Y., Liu H., Wang Z.R., Zhang K., Song S.M., Yang Q. (2021). *Streptomyces luteolifulvus* sp. nov., a novel actinomycete isolated from soil in Nanjing, China. Antonie Van Leeuwenhoek.

[19] Wang L.Y., Xing M.Y., Di R., Luo Y.P. (2015). Isolation, identification and antifungal activities of *Streptomyces aureoverticillatus* HN6. J. Plant Pathol. Microbiol..

[20] Kaur T., Manhas R.K. (2014). Antifungal, insecticidal, and plant growth promoting potential of *Streptomyces hydrogenans* DH16. J. Basic Microbiol..

[21] Meenakshi S., Hiremath J., Meenakshi M.H., Shivaveerakumar S. (2024). Actinomycetes: Isolation, cultivation and its active biomolecules. J. Pure Appl. Microbiol..

[22] Khieu T.N., Liu M.J., Nimaichand S., Quach N.T., Chu-Ky S., Phi Q.T., Vu T.T., Nguyen T.D., Xiong Z., Prabhu D.M. (2015). Characterization and evaluation of antimicrobial and cytotoxic effects of *Streptomyces* sp. HUST012 isolated from medicinal plant *Dracaena cochinchinensis* Lour. Front. Microbiol..

[23] Palla M.S., Guntuku G.S., Muthyala M.K.K., Pingali S., Sahu P.K. (2018). Isolation and molecular characterization of antifungal metabolite producing actinomycete from mangrove soil. Beni-Suef Univ. J. Basic Appl. Sci..

[24] Loqman S., Barka E.A., Clément C., Ouhdouch Y. (2009). Antagonistic actinomycetes from Moroccan soil to control the grapevine gray mold. World J. Microbiol. Biotechnol..

[25] Sholkamy E.N., Muthukrishnan P., Abdel-Raouf N., Nandhini X., Ibraheem I.B.M., Mostafa A.A. (2020). Antimicrobial and antinematicidal metabolites from *Streptomyces cuspidosporus* strain SA4 against selected pathogenic bacteria, fungi and nematode. Saudi J. Biol. Sci..

[26] Zhang J., Wang Y., Du Z.L., Lin D.S., Huo L.L., Qin L., Wang W., Qiang L.W., Yao Y.P., An Y. (2021). Screening, identification and antagonistic effect of antagonistic bacteria JTFM1001 against aflatoxin contamination in corn. Oil Crop Sci..

[27] Ahsan T., Chen J.G., Wu Y.H., Irfan M., Shafi J. (2017). Screening, identification, optimization of fermentation conditions, and extraction of secondary metabolites for the biocontrol of *Rhizoctonia Solani* AG-3. Biotechnol. Biotechnol. Equip..

[28] Lu Y.X., Song W., Wang J., Cao Y., Han X., Xu C.L., Wang F., Ge B.B. (2024). Biocontrol of *Botrytis cinerea* by *Streptomyces noursei* C27 and preliminary identification of antimicrobial metabolites. Biol. Control.

[29] Adnani M., El Hazzat N., El Alaoui M.A., Selmaoui K., Benkirane R., Touhami A.O., Douira A. (2024). In vitro and in vivo study of the antagonistic effects of a Trichoderma strain against four isolates of Fusarium that are pathogenic to chickpea. 3 Biotech.

[30] Chen N.C. (1991). Pesticide Bioassay Technology.

[31] Ren L., Wang S.F., Shi X.J., Cao J.Y., Zhou J.B., Zhao X.J. (2020). Characterisation of sensitivity of *Colletotrichum gloeosporioides* and *Colletotrichum capsici*, causing pepper anthracnose, to picoxystrobin. J. Plant Dis. Prot..

[32] Li N., Chen S.M., Yan Z.Q., Han J.H., Ta Y.Q., Pu T.X., Wang Y.H. (2021). Antimicrobial activity and identification of the biosynthetic gene cluster of X-14952B from *Streptomyces* sp. 135. Front. Microbiol..

[33] Kelly K.L. (1964). Inter-Society Color Council-National Bureau of Standards Color Name Charts Illustrated with Centroid Colors.

[34] Shen J., Zhang C., Zhang S.Y., Chen F., Pei F., Zhou S., Lin H.P. (2022). Screening, isolation and mechanism of a nematicidal extract from actinomycetes against the pine wood nematode *Bursaphelenchus xylophilus*. Heliyon.

[35] Fatima A., Aftab U., Shaaban K.A., Thorson J.S., Sajid I. (2019). Spore forming Actinobacterial diversity of Cholistan Desert Pakistan: Polyphasic taxonomy, antimicrobial potential and chemical profiling. BMC Microbiol..

[36] Zhang R.W., Han X.X., Xia Z.F., Luo X.X., Wan C.X., Zhang L.L. (2017). *Streptomyces luozhongensis* sp. nov., a novel actinomycete with antifungal activity and antibacterial activity. Antonie Van Leeuwenhoek.

[37] Pridham T.G., Gottlieb D. (1948). The utilization of carbon compounds by some actinomycetales as an aid for species determination. J. Bacteriol..

[38] Gordon R.E., Barnett D.A., Handerhan J.E., Pang C.H. (1974). *Nocardia coeliaca*, *Nocardia autotrophica*, and the Nocardin Strain. Int. J. Syst. Evol. Microbiol..

[39] Chun J., Lee J.H., Jung Y., Kim M., Kim S., Kim B.K., Lim Y.W. (2007). EzTaxon: A web-based tool for the identification of prokaryotes based on 16S ribosomal RNA gene sequences. Int. J. Syst. Evol. Microbiol..

[40] Tamura K., Stecher G., Kumar S. (2021). MEGA11: Molecular evolutionary genetics analysis version 11. Mol. Biol. Evol..

[41] Felsenstein J. (1985). Confidence limits on phylogenies: An approach using the bootstrap. Evolution.

[42] Kageyama A., Suzuki S., Yazawa K., Nishimura K., Kroppenstedt R.M., Mikami Y. (2004). *Nocardia aobensis* sp. Nov., isolated from patients in Japan. Microbiol. Immunol..

[43] Pan J.M., Geng X.S., Cai Y.J., Yu Y., Hou Y.R., Liu Y., Ya C.N., Liu Q. (2024). Identification, fermentation optimization, and biocontrol efficacy of actinomycete YG-5 for the prevention of *Alternaria* leaf spot disease in star anise. Sci. Rep..

[44] Xu Z.Y., Lu H.L., Shi W.B., Zhou X.M., Ren J.X., Zhang Y.L., Ma R. (2024). Optimization of fermentation and biocontrol efficacy of *Bacillus atrophaeus* XHG-1-3m2. Microorganisms.

[45] Yun T.Y., Feng R.J., Zhou D.B., Pan Y.Y., Chen Y.F., Wang F., Yin L.Y., Zhang Y.D., Xie J.H. (2018). Optimization of fermentation conditions through response surface methodology for enhanced antibacterial metabolite production by *Streptomyces* sp. 1-14 from cassava rhizosphere. PLoS ONE.

[46] Gajaraj B., Nadumane V. (2020). Caspase mediated cytotoxicity of a yellow pigment produced by *Exiguobacterium alkaliphilum* on human cancer cell lines. J. Pharm. Pharmacogn. Res..

[47] Thumar J.T., Dhulia K., Singh S.P. (2010). Isolation and partial purification of an antimicrobial agent from halotolerant alkaliphilic *Streptomyces aburaviensis* strain Kut-8. World J. Microbiol. Biotechnol..

[48] Kalam S., Basu A., Ahmad I., Sayyed R.Z., El-Enshasy H.A., Dailin D.J., Suriani N.L. (2020). Recent understanding of soil acidobacteria and their ecological significance: A critical review. Front. Microbiol..

[49] Subramani R., Aalbersberg W. (2013). Culturable rare *Actinomycetes*: Diversity, isolation and marine natural product discovery. Appl. Microbiol. Biotechnol..

[50] Kang P.Z., Wu X.H., Zhang L.R. (2015). Study on inhibition methods to miscellaneous microorganism for soil actinomycetes isolation. North. Hortic..

[51] Si M.R., Xue Q.H., Lai H.X. (2004). Studies on selection of the isolation medium for actinomycetes and inhibition methods to miscellaneous microorganism. Microbiol. China.

[52] Yang Y.R., Xu L.H., Li Q.R., Jiang C.L. (1995). A study on isolation methods of actinomycetes. Microbiol. China.

[53] Chang T.L., Huang T.W., Wang Y.X., Liu C.P., Kirby R., Chu C.M., Huang C.H. (2021). An actinobacterial isolate, *Streptomyces* sp. YX44, produces broad-spectrum antibiotics that strongly inhibit *Staphylococcus aureus*. Microorganisms.

[54] Kaewkla O., Franco C.M.M. (2017). *Streptomyces roietensis* sp. nov., an endophytic actinobacterium isolated from the surface-sterilized stem of jasmine rice, *Oryza sativa* KDML 105. Int. J. Syst. Evol. Microbiol..

[55] Law J.W., Ser H.L., Duangjai A., Saokaew S., Bukhari S.I., Khan T.M., Ab Mutalib N.S., Chan K.G., Goh B.H., Lee L.H. (2017). *Streptomyces colonosanans* sp. nov., a novel actinobacterium isolated from Malaysia mangrove soil exhibiting antioxidative activity and cytotoxic potential against human colon cancer cell Lines. Front. Microbiol..

[56] Duan Y.J., Chen J., He W., Chen J.J., Pang Z.C., Hu H.G., Xie J.H. (2020). Fermentation optimization and disease suppression ability of a *Streptomyces* ma. FS-4 from banana rhizosphere soil. BMC Microbiol..

[57] Lee J.A., Kim H.U., Na J.G., Ko Y.S., Cho J.S., Lee S.Y. (2023). Factors affecting the competitiveness of bacterial fermentation. Trends Biotechnol..

[58] Kronheim S., Solomon E., Ho L., Glossop M., Davidson A.R., Maxwell K.L. (2023). Complete genomes and comparative analyses of *Streptomyces* phages that influence secondary metabolism and sporulation. Sci. Rep..

[59] Vurukonda S.S.K.P., Giovanardi D., Stefani E. (2018). Plant growth promoting and biocontrol activity of *Streptomyces* spp. as endophytes. Int. J. Mol. Sci..

[60] Briceño G., Fuentes M.S., Saez J.M., Diez M.C., Benimeli C.S. (2018). *Streptomyces* genus as biotechnological tool for pesticide degradation in polluted systems. Crit. Rev. Environ. Sci. Technol..

