# Supplementary material for: Screening, Identification, and Fermentation Optimization of the Antagonistic Actinomycete Strain TCS21-117 Against Botrytis cinerea
Source: Microorganisms. 2025 Feb 9;13(2):379. doi: 10.3390/microorganisms13020379 (PMC11858271; doi:10.3390/microorganisms13020379)
Supplement: Supplementary file 1 [file microorganisms-13-00379-s001.zip › microorganisms-3460559-supplementary.pdf]

Supplementary materials

Supplementary Tables

Table S1

Table S1. Location of soil samples collection.

Location	Latitude (N°)	Longitude (E°)
Jiangsu (Wuxi)	31.50	120.26
Zhejiang (Zhoushan)	30.74	122.44
Shanghai	31.65	121.56

Table S2

Table S2. Media formulations for isolation of actinomycetes.

Components	Types of media (g/L)				
	**PDA**	**GS NO.1**	**HVA**	**ISP-2**	**GYM**
Vitamin B1~6			0.0005		
KNO_3		1			
NaCl		0.5			
KCl			1.7		
$MgSO_4{\cdot}7H_2O$		0.5	0.05		
$FeSO_4{\cdot}7H_2O$		0.01	0.01		
Na_2HPO_4			0.5		
K_2HPO_4		0.5			
Potato	200				
Glucose	20			4	20
Yeast extract powder				4	
Yeast extract					4
Soluble starch		20			
Malt extract powder				10	10
Corn starch					10
$CaCO_3$			0.02		
Inositol			0.0005		
Humic acid			0.1		
Biotin			0.00025		
4-Aminobenzoic acid			0.0005		
Agar	20	20	20	20	20
RO water	1000	1000	1000	1000	1000
pH	7.2~7.4	7.2~7.4	7.2~7.4	7.2~7.4	7.2~7.4

Table S3

Table S3. Media formulations for fermentation of strain TCS21-117.

Components	Types of media (g/L)				
	PDB	GS NO.1	SLM	MDP	GYM
KNO_3		1			
NaCl		0.5	4	12.5	
$MgSO_4·7H_2O$		0.5			
$FeSO_4·7H_2O$		0.01			
K_2HPO_4		0.5			
Potato	200				
Glucose	20			10	20
Yeast extract					4
Yeast powder			5		
Soluble starch		20	20		
Malt extract powder					10
Corn starch					10
$CaCO_3$			2	2	2
Soybean flour			15		
Peptone			2	3	
Millet				10	
RO water	1000	1000	1000	1000	1000
pH	7.2~7.4	7.2~7.4	7.2~7.4	7.2~7.4	7.2~7.4

Supplementary Figures

Figure S1

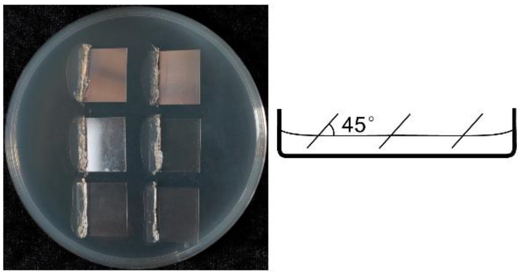

Figure S1. Schematic diagram of the insertion method.

Figure S2

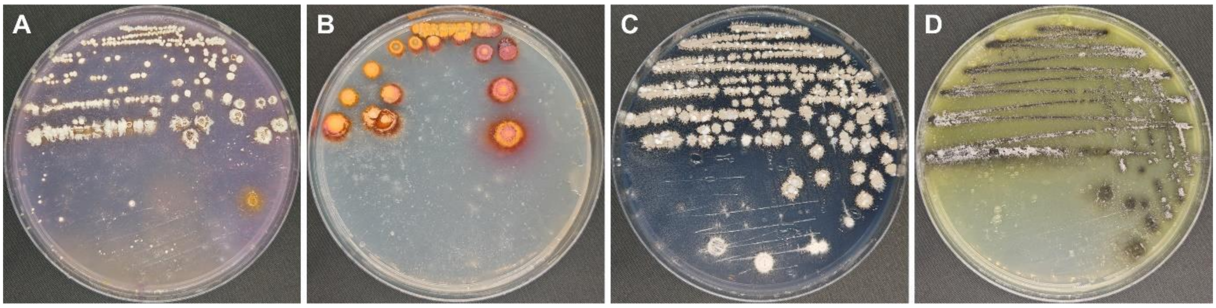

Figure S2. Special pigments produced by four *Streptomyces* isolates in this study. (A) TCS21-007; (B) TCS21-026; (C) TCS21-057; (D) TCS21-079.

Figure S3

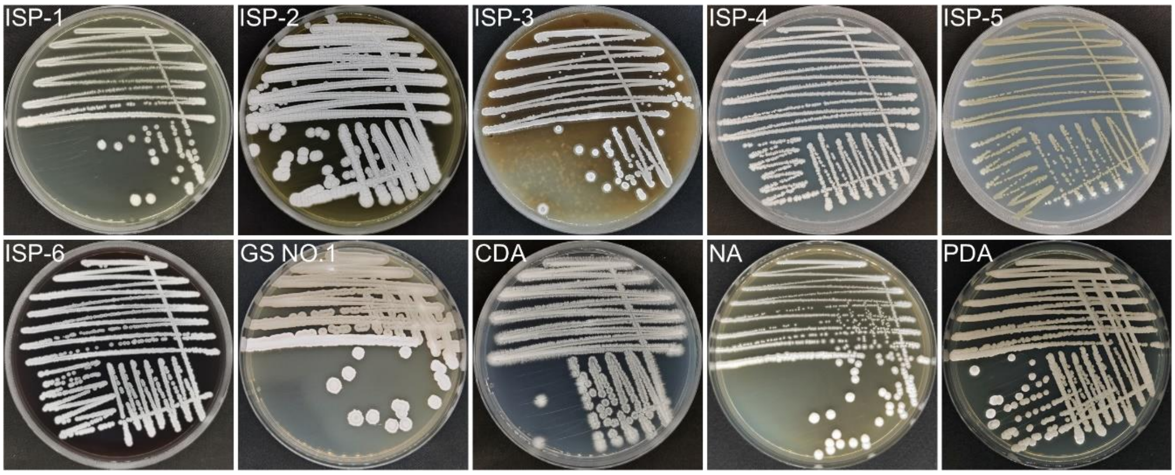

Figure S3. The colony morphology of strain TCS21-117 on different medium plates.

Figure S4

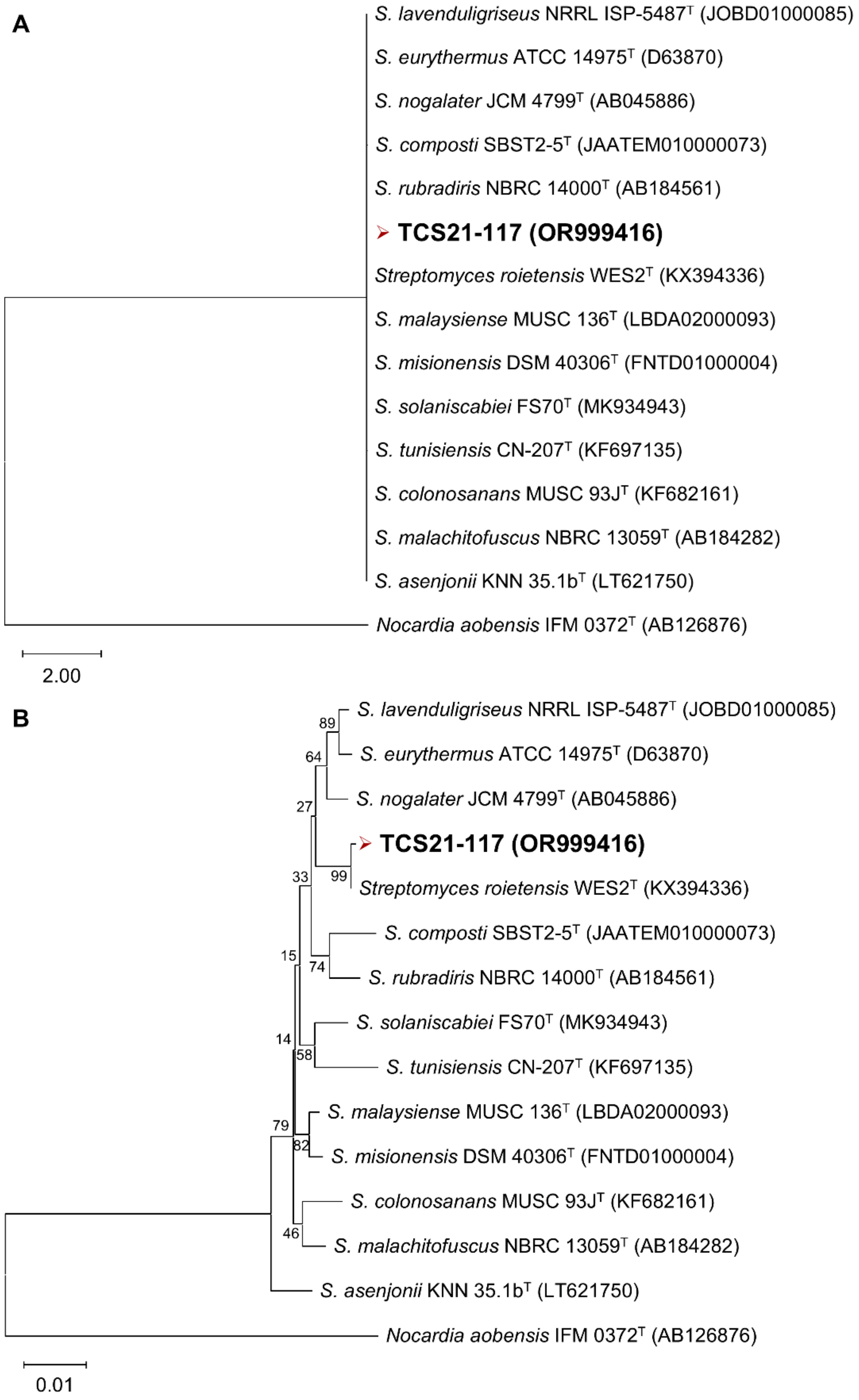

Figure S4. The result of phylogenetic analysis using the maximum-likelihood (A) and maximum-parsimony (B) tree-making algorithms.
